# Building trust and rapport early in the new doctor-patient relationship: a longitudinal qualitative study

**DOI:** 10.1186/s12909-017-0868-5

**Published:** 2017-02-02

**Authors:** Bich N. Dang, Robert A. Westbrook, Sarah M. Njue, Thomas P. Giordano

**Affiliations:** 1 VA Center for Innovations in Quality, Effectiveness and Safety (IQuESt), Houston, TX USA; 2 Michael E. DeBakey Veterans Affairs Medical Center (152), 2002 Holcombe Blvd, Houston, TX 77030 USA; 3 Department of Medicine, Baylor College of Medicine, Houston, TX USA; 4 Jesse H. Jones Graduate School of Business, Rice University, Houston, TX USA

**Keywords:** Patient satisfaction, Patient-centered care, Patient preference, Physician-patient relations, Patient engagement, Health communication, HIV infection, Retention in care, Qualitative studies, Longitudinal studies

## Abstract

**Background:**

New patients are a particularly vulnerable population because they are at high risk of missing a subsequent visit or dropping out of care completely. However, few data exist on what new patients value in the beginning of a relationship with a new provider. Persons with HIV infection may be an ideal population to study the drivers of a positive initial patient-provider relationship, as it is a chronic and serious condition that requires a reliable, ongoing relationship with a provider. Informed by patients’ real experiences, this study aims to identify what patients see as the most critical elements for building trust and rapport from the outset.

**Methods:**

We conducted longitudinal, in-person interviews with 21 patients new to the HIV clinic at the Michael E. DeBakey Veterans Affairs Medical Center in Houston, Texas, from August 2013 to March 2015. Patients were interviewed across three time points: once before their first provider visit, a second time within two weeks after the first visit, and a third time at 6 to 12 months after the first provider visit.

**Results:**

We conducted 61 h of patient interviews. The mean age was 53 years; 52% were non-Hispanic white, 23% were non-Hispanic black and 19% were Hispanic. Patients described significant anxiety and vulnerability not just from HIV itself, but also in starting a relationship as a new patient to a new provider. Our analysis of these experiences revealed five actions providers can take to reduce their patients’ anxiety and build trust early in the first visit: 1) provide reassurance to patients, 2) tell patients it’s okay to ask questions, 3) show patients their lab results and explain what they mean, 4) avoid language and behaviors that are judgmental of patients, and 5) ask patients what they want [i.e., treatment goals and preferences].

**Conclusions:**

Our study incorporates direct input from patients and highlights the unique psychological challenges that patients face in seeking care from a new provider. The actionable opportunities cited by patients have the potential to mitigate patients’ feelings of anxiety and vulnerability, and thereby improve their overall health care experience.

## Background

The patient’s first visit with a new provider is critical because it has the potential to shape attitudes and behaviors that foster and strengthen the patient-provider relationship [1–4]. Judgments made in the first few minutes of an interaction can have a major impact on a number of behavioral outcomes, e.g., the likelihood of getting a job offer, making a friend, getting a second date, and closing a sale [5, 6]. In healthcare, the first few minutes of an initial patient-provider encounter are likewise important for establishing trust and rapport. Relationships characterized by trust and rapport not only contribute to better care experiences, but they can also alleviate anxiety and distress and enhance patients’ involvement in decisions about their care [7–9]. Patients’ initial care experience can also impact behaviors such as the likelihood of taking medicines as prescribed and returning for a second visit [10, 11]. Adherence to medications and retention in care are particularly important for the proper management of chronic diseases [12–14]. In our prospective study of patients new to an HIV primary care provider, patients with better initial experiences and greater trust in the provider were significantly more likely to complete a second visit, take their medicines as prescribed and remain in care [11]. These findings suggest that investing in the first patient interaction not only has intrinsic value, but may also impact clinically relevant outcomes. However, few data exist on what new patients seek in their first visit, and how the concerns of new patients may differ from those of established patients.

New patients are a particularly vulnerable population in that they are most at risk of missing a subsequent visit or dropping out of care completely. While there are a number of chronic disease contexts in which to study the drivers of a positive initial doctor-patient interaction, we chose the care of persons with HIV infection. HIV infection is particularly well-suited to studying the patient care experience, as it is a life-altering but manageable condition that requires a reliable, ongoing relationship with the provider.

Patient-centered care is an important contributor to a positive patient care experience. A large body of literature on patient-centered communication cites trust and rapport as critical to fostering positive patient-provider relationships [15]. However, many questions remain. For example, beyond trust and rapport, what do patients say is important to them in their first and subsequent encounters with providers? As an initial inquiry into these questions, we interviewed patients new to an HIV primary care provider at multiple time points during their first 12 months of care. We were particularly interested in identifying actionable opportunities for creating a positive care experience for new patients. Informed by patients’ real experiences, this study aims to identify what patients see as the most critical elements for building trust and rapport from the outset.

## Methods

### Study population

This study took place within the context of a larger qualitative study seeking to understand how patients anticipate, experience and evaluate their HIV care. We conducted longitudinal, in-person interviews with 21 patients new to the HIV clinic at the Michael E. DeBakey Veterans Affairs (VA) Medical Center in Houston, Texas. This clinic is the third largest HIV clinic within the Department of Veterans Affairs, caring for almost 1,000 patients yearly. Patients were new to the HIV clinic at the DeBakey VA, but were not necessarily newly diagnosed and may have been treated elsewhere in the past. Eligible patients: 1) were age 18 years or older; 2) had confirmed HIV infection; and 3) had not completed an HIV provider appointment at the DeBakey VA prior to enrollment.

The Institutional Review Board at Baylor College of Medicine and the DeBakey VA Research and Development Committee approved this study. All participants gave written informed consent. To protect their confidentiality, all names used in the text are pseudonyms.

### Development and pre-testing of the interview guide

The study team developed a semi-structured open-ended interview guide to elicit information about patients’ ideals, hopes, expectations and evaluations of the HIV provider (see Table 1). We pilot tested the interview guide with five established patients at the DeBakey VA HIV clinic. We conducted cognitive interviews, using the Think Aloud method [16], from July 30 to August 13, 2013. Cognitive interviews ensured that questions and probes were easy to understand and elicited relevant data. Participants received $20 in compensation for the one-time interview. Cognitive interviews lasted on average 60 min and were audio-recorded and professionally transcribed verbatim. Based on patient feedback, we made content and wording revisions to the interview guide.

**Table 1:** Major topics and key questions, according to interview time point

Pre-visit	Now let’s talk specifically about your HIV doctor. For your upcoming visit:	
	Hopes	Your first HIV clinic visit is coming up. What’s been going through your mind? What do you hope you’ll get from that visit? How would you know your doctor is providing you the best HIV care? For you personally, what would make your doctor a perfect match for you and your needs? How would he or she work with you, talk to you, treat you?
	Expectations	What do you think your HIV doctor will do? What do you think your doctor will talk about?
	Past experiences	How do you think this doctor will compare to other doctors that you’ve seen in the past for other things? The other doctors can be any type of doctor.
0–2 weeks post-visit	Last time we talked about your plans and expectations. Today, I would like to focus on how your visit actually went. First, let’s talk about your HIV doctor.	
	First impressions	What was your first impression of the doctor? How different or similar was the doctor from what you thought he/she would be like?
	Valued attributes	What did you like about the doctor? What did you not like about the doctor? Using any number from 0 to 10, where 0 is the worst doctor possible and 10 is the best doctor possible, what number would you use to rate your doctor? What did you have in mind when you gave a ___ rating? What would make you give a 10 rating? Assuming you can switch doctors, what are some things that would cause you to switch to another HIV doctor at this clinic?
	Actionable opportunities	Is there anything the doctor could have done to make your experience better? Did the doctor tell you everything you needed or wanted to know? If you could change one thing about your HIV doctor, what would you change?
6–12 months post-visit	Last time we talked about how your first visit to the HIV clinic went. Today I’d like to talk about what’s gone on since that first visit. Tell me about your most recent visit with the HIV doctor.	
	Journey in patient-provider relationship	Think about how you felt right after your first visit with the doctor and how you feel right now about the doctor. Has anything changed for you?
	Valued attributes	What did you like about your most recent visit with this doctor? What did you not like about your most recent visit with this doctor? Using any number from 0 to 10, where 0 is the worst doctor possible and 10 is the best doctor possible, what number would you use to rate your doctor? What did you have in mind when you gave a ___ rating? What would make you give a 10 rating?
	Actionable opportunities	Is there anything the doctor could have done to make your experience better? Did the doctor tell you everything you needed or wanted to know? If you could change one thing about your HIV doctor, what would you change?

### Longitudinal qualitative study

We conducted a longitudinal qualitative study to understand how patients’ expectations and evaluations of their provider develop and change over time. We interviewed patients at three time points: once before their first HIV provider visit, a second time within two weeks after the first visit, and a third time between 6 and 12 months after the first HIV provider visit. The first interview focused on the patient’s past experiences with clinical care and the patient’s ideals, hopes, and expectations of the HIV provider. The second interview gathered the patient’s first impression of the HIV provider. The third interview examined how the patient’s evaluation of the HIV provider evolved over time, presumably after having had repeated visits with the same provider.

Patients referred to the HIV clinic were contacted by clinic staff to schedule their first appointment with the HIV provider. At that time, eligible patients were queried about their willingness to be contacted by study staff to take part in a research study. Patients granting permission were invited to participate in the study. Recruitment and interviewing continued until we reached data saturation [17].

Recruitment took place from August 2013 to July 2014. The Principal Investigator (PI, BND), an infectious diseases physician with postdoctoral training in health services research, and a study coordinator (SMN), a Masters level public health professional with a background in health promotion and behavioral science, conducted the in-person interviews. Interviews took place in private rooms at the VA or locations convenient for participants, such as libraries and college campuses. Interviews lasted an average of 61 min, and were audio-recorded and professionally transcribed verbatim. Participants received $10 in compensation for the first interview, $15 for the second interview, and $25 for the third interview.

### Data analysis

Transcribed interviews were imported into Atlas.ti software for data management. Interviews were coded using directed and conventional approaches to content analysis [18]. Coding categories were derived from existing literature on the patient experience and patient-provider relationship (e.g., directed approach) and the data itself (e.g., conventional approach). Impressions recorded during and immediately after each interview and key concepts that emerged from close reading of the first set of transcripts were used to identify additional codes. The full research team then reviewed the list of preliminary codes, and through discussion, further refined and revised the codes. A final list was developed of the codes and their definitions and explicit guidelines for use. Two researchers (BND and SMN) independently listened to all the recordings and coded all of the interviews. The two researchers met regularly to compare codes, and discuss and resolve differences. Interview text was analyzed at each time point (i.e., at each time point, data from all patients were analyzed together as a group) and longitudinally (i.e., data from each patient were analyzed across time) [19, 20]. Once all the data had been coded, query reports were generated from all the codes related to the patient’s experience with their provider. The team reviewed the query reports, writing analytic memos to identify patterns. A second round of more in-depth analyses focused on: 1) how patients feel and think as they approach a new provider, and 2) action oriented steps that providers can directly translate into clinical practice. We define key themes below.

## Results

Twenty one patients completed the pre-visit interview, 20 completed the initial post-visit interview and 16 completed the third interview at 6–12 months after the first HIV provider visit. We conducted 61 h of patient interviews. The mean age was 53 years (range 25–76) and the majority were men (*n* = 19). The group represented a diverse mix of racial and ethnic groups (11 non-Hispanic white, five non-Hispanic black, four Hispanic, and one Asian patient). Most were new to the Department of Veterans Affairs health care system (*n* = 15), four were transferring HIV care from another VA medical center and one was already enrolled at the DeBakey VA. One patient was newly diagnosed with HIV.

### Patients assume the provider is knowledgeable; what they hope for is a provider who genuinely cares

When asked about whether they had any expectations of the new provider, patients typically said they were “unsure” what to expect and did not want to expect too much. For the most part though, patients assumed that their new doctor would be knowledgeable. During his first interview, prior to meeting his new provider, Rob, a mechanic in his late-40s, says:

I always thought the first specialist that I seen, I thought that [he] was the only one that knew what was going on. Then after [I] seen some others, I figured out that they’re all pretty much educated and that’s why they’re called a specialist because they know in-depth about the disease.

When speaking to Rob at his third interview, he restated his assumptions, drawing on his prior experiences with providers at other clinics.

I’ve had three really good doctors and one of them was uh everyone said he’s the best in Houston. And then I had another one after him and I’m like, “Well he’s just as good as he.” Then I went to [another doctor, non-VA] and I thought she was just as good as the other two. And so I’m thinking you know they went to the same school.

Rather than focusing on the provider’s knowledge, patients mostly talked about their hopes for a compassionate provider who would address their concerns. George, a man in his early-40s who just moved to Houston for a banking job, says during his first interview:

I hope I’ll get a sense of, some kind of sense of security… that, you know, I’m gonna be taken care of.

### Patients experience a lot of anxiety and vulnerability as a new patient to a new provider

Patients described anxiety and vulnerability stemming not only from their HIV diagnosis, but also from being a new patient to a new provider. Joe, a man in his early-50s who wants to leave his job as an analyst and pursue his passion in photography says during his second interview, 12 days after his first visit with the provider:

I just think that finding a doctor period puts you in a place where you’re vulnerable… part of moving to the VA was it’s free and that’s a good thing but I knew… I was going to take a risk of finding a good doctor equal to or better than my current doctor….

Importantly, Joe’s feelings of vulnerability remained and were still evident in his third interview, six months after his first visit with the provider. He says:

Face it, you go to the doctor [when] you’re in a complete state of vulnerability. There’s something wrong with you and you need someone to tell you you’re okay or you’re going to be okay. And if the doctor just dismisses you and says, “You know and I’ve got better things I need to do right now,” the last thing on your mind is ever going back to that doctor.

Patients wanted and needed to believe that their new doctor would have their best interest at heart. This was a particularly salient point for patients who already had a doctor they liked, but who had to switch doctors for financial or relocation reasons. Patients also talked about their fears of rejection and/or apathy by the new provider, one who may “dismiss” their health concerns and worries. Both sentiments speak to the psychological reality inherent in seeking treatment in a state of vulnerability and anxiety.

For some, the idea of having to rehash their HIV story to a new doctor was particularly anxiety-provoking. Even patients with a longstanding diagnosis of HIV reported a fear of being questioned and forced to relive the story of how they were diagnosed. For these patients, having to again describe how they contracted the disease elicited negative emotions including anger, guilt, shame and severe anxiety. Ken, a retired electrical lineman in his late-60s, whose wife is the only person who knows of his diagnosis, says in his first interview:

I don’t want to have to sit there and every time go through the same thing over to the next doctor and the next doc…. ‘How did you get this disease?’ ‘Why did you get this disease?’ ‘How long have you had this?’ I don’t want to discuss that anymore. It’s done and over with.

Instead, these patients want to focus on knowing the status of their HIV and what they need to do to stay healthy. Patients reported that questions like, “Do you have sexual relations with men or women?” to assess risk factors are not a problem. However, questions such as “How did you get HIV?” provoke negative emotions.

### First impressions matter and continuity of care is important to patients

Patients’ first impressions of the care received at their first provider visit were overwhelmingly favorable, despite the anxiety and vulnerability many felt beforehand. Longitudinal analyses across the three points in time show that first impressions appear to matter. Patients formed their impressions of providers early, in connection with the initial visit, and no new themes emerged at the third interview. Patients also spoke to the importance of continuity of care and not wanting the burden of having to retell their story yet again. When asked how he would feel if for some reason he had to switch providers, Peter, a man in his early-50s recently diagnosed with HIV, felt uneasy and notes during his third interview:

With a new doctor I’d have to go back and start completely over and I don’t know if I would feel as comfortable with a new doctor as I feel with him [current VA provider].

Rick, a man in his mid-50s, was the only patient in this study who saw a different provider on his second visit. He says during his third interview:

[I] feel like I’m [being] passed around…. I didn’t get to see the same doctor so it was like starting over. Send me to somebody that’s… going to be here next time… or if they’re leaving just tell the patient that they’re moving [ahead of time]…. So I’m going to see you this time, but I won’t be seeing you next time.’

This patient had not been told in advance about the change in provider, and as a result felt disrespected and exposed.

### Actionable things providers can do to build trust and rapport

Patients identified a variety of positive experiences with the provider that were of particular value in dealing with their feelings of anxiety and vulnerability. Our analysis of these experiences revealed five actions providers can take to reduce their patients’ anxiety and build trust early in the first visit: 1) provide reassurance, 2) tell patients it’s okay to ask questions, 3) show patients their lab results and explain what they mean, 4) avoid language and behaviors that are judgmental of patients, and 5) ask patients what they want [i.e., treatment goals and preferences].

### 1) Patients want their providers to provide reassurance

For patients newly diagnosed with HIV, the need for reassurance is urgent. Jim, a college student in his mid-20s who was just diagnosed with HIV before Christmas said during his second interview, two weeks after his initial visit:

I would’ve sat there till five o’clock that evening just to be seen by the doctor. I needed clarity; I needed peace of mind…. I was nervous and anxious and I was scared…. it’s a scary diagnosis.

I want to sit in front of the doctor and the doctor to tell me like, ‘You know you’re okay.’

In the face of a serious illness, Jim’s provider played a critical role in reducing his fears and the level of stress he was experiencing during his initial visit. Incidents such as these attest to the power of the provider to foster strength and resilience by simply reassuring the patient. Here, Jim describes how his provider helped him address fears of this “scary diagnosis:”

We spoke about medication and treatment and then when I told him I needed mental help, he walked [me] straight to the counselor’s office…. And so that helped me feel a little better....It changed a lot of my fears. It went away and my stress level has come down drastically since then.

Even patients who are stable on HIV medicines still need intermittent reassurance that they can control their disease and that they are not going to die. They need to hear that they are on the right track and that they are doing okay on treatment. Bill, a flight attendant in his mid-40s who was diagnosed with HIV over a decade ago says during his first interview:

Sometimes you just want to go in and get the results of your last labs and go. Other times you need to be reassured.

The consequences of not receiving the desired reassurance from the provider can be psychologically devastating. Alex, a man in his mid-50s, attempted suicide the day after he was diagnosed with HIV at a non-VA facility. During his first interview, Alex reflects on his recent diagnosis, and how he found out:

I’m Hispanic. The mindset of having to go and tell my family that I was HIV positive, it was just overwhelming …. it was just the way the doctor presented it. It was, “I don’t care….I don’t want to be bothered with you.” ….He just said, “Oh I have no idea what, what, anything about HIV. You’re just going to have to go to infectious disease.” …It was so cold.

What I needed at the time was somebody, “Okay he’s HIV; let’s give him a doctor that knows what they’re talking about to sit down and explain what’s going on.” And then…. he wasn’t empathetic and he didn’t care you know. He just brushed me off.

This patient’s misbeliefs about HIV fueled his anxiety. He was terrified of ending “up in the hospital with blisters” and that the medicine would be “worse than the illness.” This patient needed a provider who could provide basic information and reassure him that with treatment he was not going to die prematurely. Such a conversation would have helped the patient clearly understand what to expect, which in turn, would have likely lessened his desperation.

### 2) Patients feel anxious asking their providers questions; they want their providers to tell them it’s okay to ask questions

Many patients are mindful of the doctor’s time pressures and worry about taking up too much of the doctor’s time. They do not want to feel like a burden to the provider, or be seen as a “difficult patient.” Tom, a business owner in his mid-60s, says during his first interview:

You can tell by looking at the patient waiting room and you’re back there in a room and there are people in other rooms and there’s the doctor. And whether they make you feel like it or not, you don’t want to tie up too much of their time because you saw all those people sitting in the waiting room and he’s got to see all of them, so you get the feeling that he’s in a rush.

Other patients worry that asking questions may offend their doctors. They worry doctors will feel that their expertise is being challenged. When they do ask questions, and the experience is favorable, patients feel relieved and highly appreciative. Peter, who underwent a lumbar puncture, felt great relief when the doctor addressed his concerns in a receptive manner. He describes his interaction with the provider during his third interview:

I just asked her. I said, “Well ma’am let me ask you question. How much experience do you have doing this?” She said, “I’ve done it before.” And she started laughing because I asked her that question, because when you start talking about spines that- that made me nervous because you know you can get paralyzed very easily. So I just had a question and she didn’t get offended by me asking the question….. Even the residents that came in were easy to talk to and easy to ask questions. And I think that’s the biggest thing when you see a doctor; not being afraid to ask a question.

Even though the doctor provided only a minimal response (by saying she had performed the procedure at least once), the patient was accepting, and moreover happy that he had not offended the doctor.

Many patients want the doctor to explicitly state that say it’s okay to ask questions. Tom, who rated his doctor a 10 out of 10, gave an example of how his doctor made him feel comfortable asking questions. During his second interview, he says:

After going through different parts with him he’d say, ‘Have any questions about that?’ And then a little later, ‘Do you have any questions about that?’ ….And then again he summarized it as, ‘Are there any other concerns or questions or anything else we need to address today?’

This patient particularly liked having his doctor ask if he had any questions at multiple points in the visit, rather than only at the end of the visit. Repeated invitations to ask questions allowed the patient to get answers for all the issues of concern, as well as to ask some that he would not have otherwise felt comfortable asking.

### 3) Patients want to see their lab results and for the doctor to explain what they mean

Patients described anxiety over whether or not their HIV virus was still undetectable. For those patients, simply being told that their labs “looked good” was not enough. They want to see their actual test results on the computer screen or print out, and be told if the numbers look better or worse. David, a former occupational health technician in his mid-30s, explains the importance of knowing these details:

That’s one thing that a lot of medical care professionals do. They have a habit of forgetting, you know, you do seventeen vials of blood. They- I want to know what those seventeen vial- vials of blood are going for.

Jane, a retired graphics artist in her early-60s, compared a provider who went over her labs to one who did not. She said during her first interview:

She was so nice. She’d take your screen monitor and she’d go over my T cells and all of the blood work…. And the next one didn’t and I had to call my nurse…. But I shouldn’t have to ask….I think they [doctors] should automatically discuss…..don’t just look at the numbers for your own needs…. Explain to me what these numbers are.

Another patient Rick echoed Jane’s point and explains during his third interview:

I’m not dumb. Actually I have a very high IQ….Turn the screen around or hand me a printout or something.

Patients, especially those with HIV, derive meaning from learning the specifics of their lab work, which indicate how they are doing in their overall health and tell them what they need to change to live a long and healthy life.

### 4) Patients do not want to feel judged by their providers

Whether it’s about their lifestyle or diet, patients do not want their providers to use language or behaviors that are judgmental. When doctors respond kindly and without judgment, patients take particular note. John, a computer engineer in his late-40s, says during his second interview:

[He was] empathetic to uh you know, accepted my relationship with Carlos and that we had kids. There was no uh- uh you know off color remarks… nothing like that.

Chris, a retired pharmacy technician in his mid-60s, worried that his doctor would scold him about his weight and eating habits. He describes in his second interview how his doctor reacted to his high cholesterol:

And she told me about my cholesterol being high. She says well it's 200. Anyway, she didn’t scold me or anything, which was nice…. I felt comfortable.

Patients want above all a supportive doctor, one who does not point out shortcomings that they already are aware of. Rather, they want a doctor who will make a genuine effort to understand them.

### 5) Patients want to be participants in medical decision-making; they want providers to ask them what they want [i.e., treatment goals and preferences]

Patients value their doctors’ knowledge and recommendations. However, they still want to be asked “So what do you think about that plan of treatment?” For example, some HIV medication regimens are complicated and most patients do not want to choose their regimen. However, they still want the doctor to lay out their options and ask for their input. Tim, a man in his early-50s who was just diagnosed with HIV, says in his first interview:

Give me all my options, lay them out there and let me see what’s up. And then try to work with me in that regard.

In his second interview, Ken the retired electrical lineman, talked about a positive experience with a doctor who explained the rationale for his recommendations and made sure Ken agreed with them:

He didn’t just say, “I’m going to change this to this.” You know he….told me why…. and what the change in the medicine [was].

Patients want a two way dialogue with their provider, an interactive exchange where patients are asked about their treatment goals and preferences. They want to feel like they have a say, and that the doctor is willing to work with them in achieving those goals. In essence, patients want their doctors to involve them in decisions about their care.

## Discussion

This qualitative study provides a strong understanding of what patients value in their health care providers. Our analyses show that patients experience significant anxiety and vulnerability not just from HIV itself, but also in starting a relationship as a new patient to a new provider. Our study is unique in identifying five actionable behaviors that have the potential to greatly improve the patient care experience: 1) provide reassurance, 2) tell patients it’s okay to ask questions, 3) show patients their lab results and explain what they mean, 4) avoid language and behaviors that are judgmental of patients, and 5) ask patients what they want [i.e., treatment goals and preferences]. Patients cited these items as effective in mitigating their anxiety and building a trusting, long-term relationship with the provider.

Patients entering a new relationship with a provider can experience heightened psychological distress, ranging from feelings of vulnerability as a new patient, to fears, situational anxiety and panic, especially when the condition is life-altering. Moreover, emotional needs may differ markedly between new patients recently diagnosed with a life-altering illness versus those who have experienced the illness for some time, and those who encounter greater illness intrusiveness [21]. A key step in building a therapeutic relationship is to recognize that some patients may have greater emotional needs, and that one approach may not fit all [22, 23]. In fact, providers who recognize this may be more attuned to emotional cues and listen more deeply to new patients’ underlying concerns [7].

Our qualitative findings show that many patients want to play an active role in their own medical care. They want to engage in a two-way dialogue with their provider, clarify expectations, voice their concerns and ask questions. At the same time, however, many patients do not see the doctor’s office as a safe place to have those conversations. They are reluctant to ask questions for fear of being perceived as a difficult patient [24]. Even highly educated patients and patients with a medical background worry about the consequences of asking questions. These beliefs, compounded with the inherent power differential between patient and doctor, cause patients to fear asking even valid questions like, “How many times have you done this procedure?” Instead, patients avoid questions that they think might be seen as contentious for fear that their care will suffer. Strikingly, even patients with a serious medical condition like HIV infection, where the stakes are far greater, worry about offending their doctor. This “white-coat silence” phenomenon is a major barrier to empowering patients to ask questions [25]. In the absence of the doctor explicitly reassuring them that it really is okay to ask questions, many patients remain silent. To facilitate open communication, patients want doctors to give them explicit permission and encouragement to ask questions.

Our data suggest that although patients certainly value affective reassurance (e.g., communication that creates rapport and conveys empathy), they derive as much if not more value from cognitive reassurance (e.g., clear explanation of their condition and treatment plan) [26–31]. Many patients in our study did not want to hear only that they were “going to be okay” or that their “labs look good.” Our study adds to the literature, highlighting the therapeutic effect of literally showing patients their test results and clearly explaining what each of the test results mean. In our study, these actions not only reassured patients, they also empowered and encouraged them to become more proactive in managing their condition.

Patients with stigmatizing conditions such as HIV infection, mental illness, substance abuse and obesity, worry about their providers judging them. Our data show how providers, even with good intentions, may inadvertently ask patients questions that elicit feelings of blame, shame and anxiety (e.g., “How did you get HIV?”). Research indicates that stigma in the health care setting is still quite prevalent, and can lead providers to spend less time with the patient, have lower expectations of their adherence, and make fewer referrals for preventive and specialty care [32–38]. Our findings underscore the importance of recognizing implicit and explicit biases that may negatively affect the quality of the patient encounter, and undermine the provider’s own desire to provide quality care. Perspective-taking exercises, where providers practice taking deliberate steps to understand their patients’ perspectives, can increase awareness of unconscious bias, increase empathy and overcome attitudes that negatively impact clinical expectations and decision making [39, 40].

Despite the clear benefits of patient-centered care, time pressure and lack of training in communication skills are structural challenges facing providers. Physicians have to balance spending enough time with each patient and staying on schedule. To achieve this balance, physicians may feel forced to interrupt patients and minimize open-ended questions. In the classic 1984 study by Beckman and Frankel, doctors interrupted patients, on average, 18 s after they started stating their concerns [41]. A more recent study confirmed these results [42]. In instances where doctors did not interrupt, patients completed what they had to say in 32 s. Interestingly, family physicians with training in communication skills were significantly less likely to interrupt (44% vs 22%, *P* = 0.012). Training in communication skills, such as up-front agenda setting, can also help providers prioritize concerns and decrease “by-the-way” questions at the end of a patient encounter (e.g., “Before we start, I’d like to make a list of everything you want to talk about today.”) [43–47]. Other communication skills such as conveying empathy and providing reassurance do not necessarily extend the visit length but can greatly enhance the quality of the patient-provider relationship [48, 49].

Our study points to strategies health care organizations can implement with minimal costs or changes to clinic flow. Given the importance of the first impression for building trust and rapport early on, care organizations can strategically schedule new patients at times of the day when providers may feel less time pressured. Moreover, given the importance of continuity of care as a significant driver of a positive patient experience, care organizations can make an extra effort to make sure that patients follow up with the same provider.

Our study points to explicit steps and language medical educators can use to teach trainees and experienced physicians, so that patients feel supported and encouraged to talk openly and honestly about their concerns and worries. Medical trainees are particularly likely to benefit from such training, as they are still developing their communication skills and have yet to establish set behaviors [50]. Experienced physicians can also learn new communication techniques, especially ones that are concrete and easy to incorporate in their clinical routine [51]. To effectively teach these skills, educators must move beyond oral presentations and written handouts, and incorporate external input, such as timely, individualized feedback based on direct observations of patient-provider encounters [52–55]. The actionable steps derived from our research could serve as a framework for such feedback.

Our study has several methodological strengths. To our knowledge, this is the first qualitative study to examine what patients new to an HIV clinic expect and value in their providers. The longitudinal nature of our study allowed us to examine recurring themes that emerged over the course of six to twelve months of subsequent care. Almost half of the participants in the study were Black or Hispanic, populations underrepresented in studies on the patient care experience.

Our study has certain limitations. We conducted our study among mostly male patients with chronic HIV infection at one public institution, and our findings may not generalize to other patient and disease populations. However, our findings add to the literature on patient experience, and we believe they are especially useful to providers who care for patients with stigmatizing chronic conditions such as mental illness, substance abuse and obesity.

## Conclusions

Our study incorporates direct input from patients and highlights the unique psychological challenges that patients face in seeking care from a new provider. Our data reveal actionable steps, informed by patients’ real experiences, that providers can take to facilitate open communication and enhance patient engagement. These steps, in turn, have the potential to mitigate patients’ feelings of anxiety and vulnerability, and thereby improve their overall health care experience.
